# Conditional survival for longer-term survivors from 2000–2004 using population-based cancer registry data in Osaka, Japan

**DOI:** 10.1186/1471-2407-13-304

**Published:** 2013-06-22

**Authors:** Yuri Ito, Tomio Nakayama, Isao Miyashiro, Akiko Ioka, Hideaki Tsukuma

**Affiliations:** 1 Center for Cancer Control and Statistics, Osaka Medical Center for Cancer and Cardiovascular Diseases, 3-3 Nakamichi 1-Chome, Higashinari-ku, Osaka 537-8511, Japan

**Keywords:** Conditional survival, Cancer registries, Relative survival, Japan

## Abstract

**Background:**

We usually report five-year survival from population-based cancer registries in Japan; however these survival estimates may be pessimistic for cancer survivors, because many patients with unfavourable prognosis die shortly after diagnosis. Conditional survival can provide relevant information for cancer survivors, their family and oncologists.

**Methods:**

We used the period approach to estimate the latest 10-year survival of 38,439 patients with stomach, colorectal, lung, breast and prostate cancer diagnosed between 1990 and 2004 and followed-up from 2000–04 in Osaka, Japan. Conditional survival is an estimate, with the pre-condition of having already survived a certain length of time. Conditional five-year relative survival of one to five years after diagnosis was calculated by site, age and stage for survivors under the age of 70.

**Results:**

Five-year relative survival for stomach cancer was 60%. Conditional five-year relative survival was 77% one year after diagnosis and 97% five years after diagnosis. This means that 97% of patients who survive five years after diagnosis can survive a further five years. Conditional five-year relative survival improved successively with each additional year that patients lived after diagnosis for stomach, colorectal and lung cancer. These figures for breast and prostate cancer were stable at high survival. Liver cancer did not show an increase in conditional five-year survival.

**Conclusion:**

Conditional five-year survival is a relevant figure for long-term cancer survivors in Japan. It is important for population-based cancer registries to provide figures which cancer patients and oncologists really need.

## Background

In recent years cancer patients have been able to survive for longer than those diagnosed a few decades ago. We usually report five-year relative survival rates after diagnosis in the annual reports of regional cancer registries in Japan. However, these survival estimates may be pessimistic for cancer survivors, because many patients with unfavourable prognosis die shortly after diagnosis.

Conditional survival analysis is a method to estimate survival rates, with the pre-condition of having already survived a certain length of time. The figures have been reported in the US and other countries, and can provide cancer survivors, their families and oncologists with more relevant information [1-11].

In 2007, the Basic Plan to Promote Cancer Control Programs of Japan was approved, following the establishment of the Cancer Control ACT of Japan in 2006. Improvement of cancer care support and information services is one of the specific goals of this program [12]. Longstanding population-based cancer registry data can provide this type of useful information to cancer survivors. We used cancer patient data from the Osaka Cancer Registry, which has a long history and covers the largest population in Japan, to report conditional survival in patients with six major cancers by age group and stage at diagnosis.

## Methods

### Data sources

**Figure 1:**
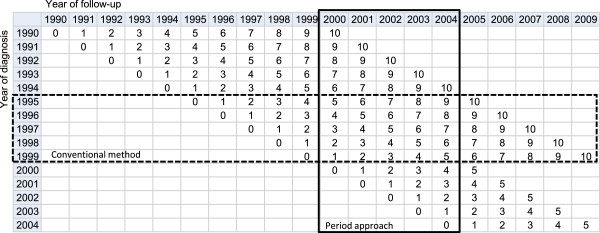
Illustration of period approach to estimate up-to-date long-term survival.

We analysed 38,439 cases diagnosed with first, primary, invasive malignant tumour of the following cancers: stomach (International Classification of Diseases, 10^th^ revision code [13]: C16), colorectal (C18-C20), lung (C33, C34), breast (C50, female only) and prostate (C61) from 1990–2004. They were followed up between 2000 and 2004 in Osaka, Japan, from the Osaka Cancer Registry database. We limited the data to patients who were diagnosed at age 15 to 69 years. Patients over 70 years of age at diagnosis were excluded from the analyses as long-term survival estimates are unstable for older age groups. In the Osaka Cancer Registry, we followed patients up using the death certificate database for residents in Osaka prefecture. However, if patients had moved outside the Osaka Prefecture after diagnosis the death certificate database would be unable to capture their details. In these cases resident registries were used to follow the patients up. Residents in Osaka-city (30% of total population of Osaka prefecture) were not followed-up using residence registries before 1993, so patients who lived in Osaka-city were excluded from the analysis. Cases diagnosed from 1990 to 1999 were followed up for 10 years after diagnosis, whereas follow-up was limited to 5 years for those diagnosed from 2000 to 2004 (Figure 1).

### Statistical analysis

First, we estimated ten-year relative survival using the Osaka Cancer Registry data by sex, age group (15–49, 50–59 and 60–69 years old) and stage at diagnosis (localised, regional and distant metastasis). Relative survival is a ratio of the observed survival (overall survival) and the survival that would have been expected if the cancer patient had only experienced the normal (background) mortality of the general population in which they live [14]. In this study we estimated relative survival using the maximum likelihood method developed by Esteve et al. [15] and the publicly available Stata programme *strel*[16]. We derived the expected survival from complete (single-year-of-age) national population life tables by sex [17]. We used conventional 10-year relative survival estimating based on data from patients who were diagnosed from1995-1999 and were followed-up over 10 years (dashed line in Figure 1). However, calculation of long-term survival using the conventional method is outdated. We therefore used period analysis to derive more up-to-date long-term survival using data from recently followed-up cancer patients [18]. In this study, we used data from cancer patients who were diagnosed from 1990–2004 and followed-up between 2000 and 2004 (solid black line, in Figure 1).

Second, we calculated conditional five-year survival for patients who survived for 0 to 5 years after diagnosis using ten-year cumulative relative survival. To estimate the five-year conditional survival of patients who survived x years, the (x + 5)-year cumulative survival rates is divided by the x-year cumulative survival. In the other words, conditional five-year survival is five-year survival of the patients who are alive several years after diagnosis [2,10].

Third, for missing stage cases, we used the multiple imputation approach [19]. We examined the characteristics of patients with missing stage before multiple imputation, then we assumed the mechanism of missingness as Missing At Random. The relative survival of the ten completed data sets contained the imputed value of stage for cases with missing information (6.4-17.1%). The imputation model was a multinomial logistic regression including follow-up time, vital status, period of diagnosis, age at diagnosis, and interactions between follow-up time and other factors. Rubin’s rules were applied to estimate relative survival and standard errors combining the ten completed data sets. All statistical analyses were performed using the standard statistical package Stata Ver. 12.1 [20].

## Results

**Figure 2:**
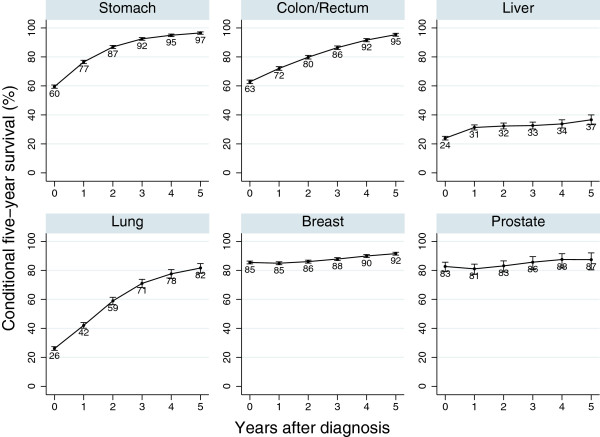
Conditional five-year survival for all patients diagnosed in 1990–2004 and followed-up in 2000–2004 in Osaka, Japan.

The characteristics of patients we analysed are shown in Table 1. Conditional five-year survival for all stage patients under the age of 70 by site of cancer is shown in Figure 2. For stomach cancer, the five-year relative survival for all cases is 60% at diagnosis. 77% of patients who survived one year (one-year survivor) can survive an additional five years. Conditional five-year survival at two years after diagnosis was 87%. Conditional five-year survival at five years after diagnosis was 97%. This means 97% of the stomach cancer patients who survived for more than five years can survive another five years. Colorectal and lung cancer showed similar results to stomach cancer patients. However, conditional five-year survival for liver cancer did not increase after any period post diagnosis. Conditional survival for breast and prostate cancer patients was stable at around 85-90%.

**Table 1:** Characteristic of cancer patients for selected sites of cancer in Osaka, Japan in 1990–2004

	**Stomach**		**Colon/rectum**		**Liver**		**Lung**		**Breast**		**Prostate**	
	***N***	**%**	***N***	**%**	***N***	**%**	***N***	**%**	***N***	**%**	***N***	**%**
**Total**	10278	100.0	8411	100.0	4800	100.0	6397	100.0	7229	100.0	1324	100.0
**Age**												
15-49	1266	12.3	897	10.7	296	6.2	549	8.6	2591	35.8	6	0.5
50-59	3442	33.5	2830	33.6	1344	28.0	2024	31.6	2752	38.1	214	16.2
60-69	5570	54.2	4684	55.7	3160	65.8	3824	59.8	1886	26.1	1104	83.4
**Stage (before imputation)**												
Localised	4863	50.7	3568	45.3	2897	72.8	1426	23.8	3747	57.3	785	65.4
Regional	2758	28.8	2577	32.7	663	16.7	2167	36.2	2439	37.3	175	14.6
Distant	1972	20.6	1728	21.9	421	10.6	2397	40.0	350	5.4	241	20.1
Missing	685	(6.7)	538	(6.4)	819	(17.1)	407	(6.4)	693	(9.6)	123	(9.3)
**Stage (after imputation)**												
Localised	5202	50.6	3817	45.4	3436	71.6	1511	23.6	4139	57.3	865	65.3
Regional	2960	28.8	2757	32.8	818	17.0	2304	36.0	2701	37.4	196	14.8
Distant	2116	20.6	1837	21.8	546	11.4	2582	40.4	389	5.4	263	19.9

### By age group

**Figure 3:**
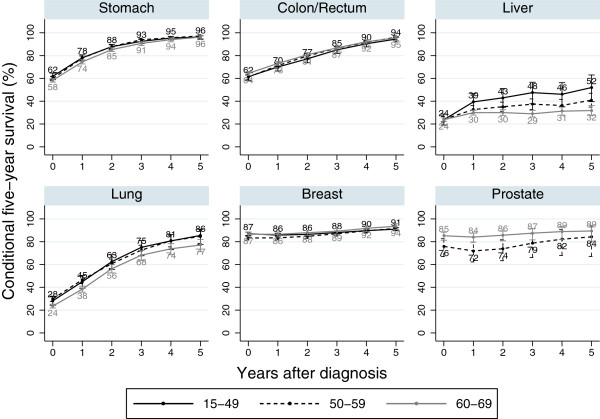
Conditional five-year survival of the patients diagnosed in 1990–2004 and followed-up in 2000–2004 in Osaka, Japan: by age group.

Results by age group are shown in Figure 3. Most cancer sites showed similar results among the different age groups. In liver cancer patients, conditional survival increased in the young group (under the age of 50). In prostate cancer patients, the older age group (60–69 years old) showed better conditional survival than the younger age group (50–59 years old).

### By stage

**Figure 4:**
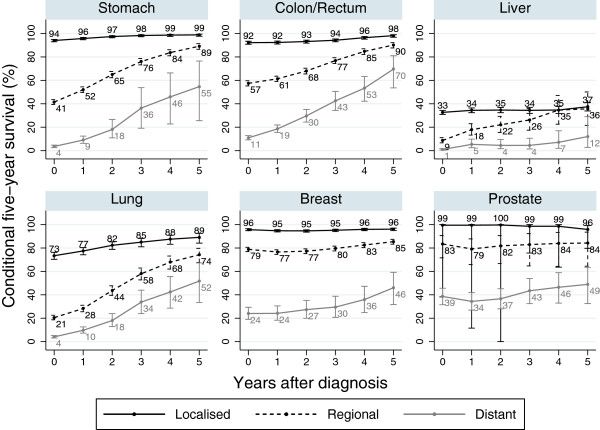
Conditional five-year survival of the patients diagnosed in 1990–2004 and followed-up in 2000–2004 in Osaka, Japan: by stage at diagnosis.

Figures for conditional survival by stage at diagnosis were different for different cancers (Figure 4). In all except liver cancer patients, the figures for conditional survival for localised patients were high at around 90%. In stomach, colorectal and lung cancer patients, even the regional and distant metastasis cases showed high five-year survival for five-year survivors. In liver cancer patients, even localised cases showed low conditional five-year survival. In breast and prostate cancer patients, conditional five-year survival for each stage was stable.

## Discussion

Conditional five-year relative survival improved successively with each additional year that patients lived following diagnosis for stomach, colorectal and lung cancer. This pattern was also similar to the regional or distant metastasis cases. Breast and prostate cancer showed different trends; conditional five-year survival was stable at a higher level. Liver cancer did not show any increase in conditional five-year survival.

### Stomach and colorectal cancer

For stomach and colorectal cancer patients who survived more than five years after diagnosis, conditional five-year survival was close to 100%. This means that those who survived five years would have the same survival probability as the general population, i.e. they can be considered as ‘cured’ of cancer. Although it is difficult to define clinical ‘cure’ at the individual level, we can define the concept of ‘cure’ at population level [21,22]. Conditional five-year survival for localised cases was stable at more than 90%. Those for regional or distant metastasis increased according to the number of years since diagnosis. Even late stage patients who survive a few years have a chance of living another five years.

### Lung cancer

Conditional survival in lung cancer patients increased according to the additional years after diagnosis. However, the figures were lower than those for stomach and colorectal cancer. This was because lung cancer patients have a higher risk of death due to complications related to cancer or cancer risk (smoking), such as ischemic heart disease.

### Breast and prostate cancer

Conditional five-year survival rates for all cases of both breast and prostate cancer were around 80-90%, due to the higher proportion of localised patients in all cases. Conditional five-year survival for localised prostate cancer patients slightly decreased five years after diagnosis. This could be partly explained by the recurrence or progression of tumours during long-term follow-up. For these cancers, we need to follow-up patients for a longer period.

### Liver cancer

Conditional survival for liver cancer was much lower than for other cancers at any stage or age after several years. Even in localised patients, conditional five-year survival was less than 40% after five years. This is probably because many liver cancer patients experienced a recurrence of cancer, or died from liver cirrhosis or liver failure related to the hepatitis B or C virus.

### Effect of age and stage at diagnosis

Trends in conditional survival by age group were quite similar except for liver and prostate cancer. For most cancers, age did not significantly affect conditional survival. In the case of liver cancer, conditional survival for young patients (15–49 years old) was higher than for old patients (60–69 years old) after several years. This could be explained by the fact that the old patients had been exposed to hepatitis viruses for long time; as a result, they tended to develop liver cirrhosis and liver failure more than young patients. Conditional survival of young prostate cancer patients (50–59 years old) was lower than old patients (60–69 years old). This is probably because young patients are diagnosed at a more advanced stage than old patients (the proportion of distant metastasis was 35.2% in 50-59-year-old patients and 28.3% in 60-69-year-old patients).

The conditional survival curve was different by stage; stage at diagnosis was an important prognostic factor. Conditional survival for localised patients was stable at 85-95%, while for regional and distant metastasis patients it increased after several years of diagnosis.

Trends in conditional survival for breast and colorectal cancer patients in Osaka were similar to other countries (shown in Additional file 1: Figures S1-S4 from Australia [2,6], US [8], Canada [5,11] and European countries [3,9]). Conditional survival for prostate cancer at all stages in Osaka was lower than other countries. This is due to the low proportion of localised patients in Japan compared to other countries. Conditional survival of stomach cancer for all stage and localised in Osaka was higher than in Australia [2]. Stomach cancer patients in Osaka were diagnosed at an earlier stage than in Australia (e.g. 51% patients diagnosed at localised stage in Osaka, 28% in Australia). In addition, approximately half of the stomach cancer patients in Japan were diagnosed at T1 (UICC TNM classification) [23]. Therefore we can estimate a higher proportion of T1 in localised patients in Osaka than in Australia. Higher conditional survival for localised patients can be partly explained by differences in tumour. This may be due to stomach cancer screening programmes [24] and wide use of endoscopy in clinical settings in Japan. Conditional survival of localised lung cancer in Osaka was higher than in other countries. This could be explained by differences in tumour size and histology [25,26]. Conditional survival for liver cancer patients in Canada increased some years after diagnosis [11], while in Osaka it was stable at low survival. This can be explained by the differences in etiological factor among these countries. In the US and Canada, prevalence of hepatitis B or C viruses in liver cancer cases was lower than in Japan [27]. Liver cancer patients in Japan might have greater likelihood of liver failure or hepatitis-related cirrhosis than those in the US and Canada.

Conditional five-year survival for stomach and colorectal cancer patients who were alive five years after diagnosis was about 100%; this means those patients have similar survival probability to the general population. Therefore those patients can be considered as ‘cured’. For other sites of cancer, further long-term follow-up time may be needed to estimate ‘cured’ time.

Conditional survival is an important statistic for planning long-term life after diagnosis, not only for cancer patients and their families, but also patients with other diseases. However, a population-based disease registry system, such as the population-based cancer registry, is essential to estimate this type of statistic.

## Conclusion

Conditional five-year survival is a relevant figure for long-term cancer survivors in Japan. It is important for population-based cancer registries to provide figures which cancer patients and oncologists really need.

## Competing interests

The authors declare that they have no competing interests.

## Authors’ contributions

YI, TN and HT developed the study concept. YI and IA were responsible for data management and statistical analysis. TN, IM and HT reviewed the clinical background of the results. YI wrote the draft of the report. All authors critically reviewed and revised the manuscript.

## Pre-publication history

The pre-publication history for this paper can be accessed here:

http://www.biomedcentral.com/1471-2407/13/304/prepub

## Supplementary Material

Additional file 1: Figure S1-S4 Comparison of conditional survival between countries, by site and stage. Click here for file
