# Supplementary material for: Conditional survival for longer-term survivors from 2000–2004 using population-based cancer registry data in Osaka, Japan
Source: BMC Cancer. 2013 Jun 22;13:304. doi: 10.1186/1471-2407-13-304 (PMC3701478; doi:10.1186/1471-2407-13-304)
Supplement: Additional file 1: Figure S1-S4 — Comparison of conditional survival between countries, by site and stage. [file 1471-2407-13-304-S1.pdf]

Additional figures:

Comparison of conditional survival between countries, by site and stage

Figure S1. All stages
Figure S2. Localised
Figure S3. Regional
Figure S4. Distant

Osaka: Our data

US: [8] Merrill RM et al. Oncologist 2010

Canada: [5] Ellisson LF et al. Health Rep 2011.

AUS QL (Australia Queen Land): [6] Baade PD et al. Med J Aust 2011.

AUS NSW (Australia, New South Wales): [2] Yu XQ et al. BMC cancer 2012.

*In the colon/rectum figures, the dashed line represents rectum results and the solid line represents colon results (as some papers provide the results separately)

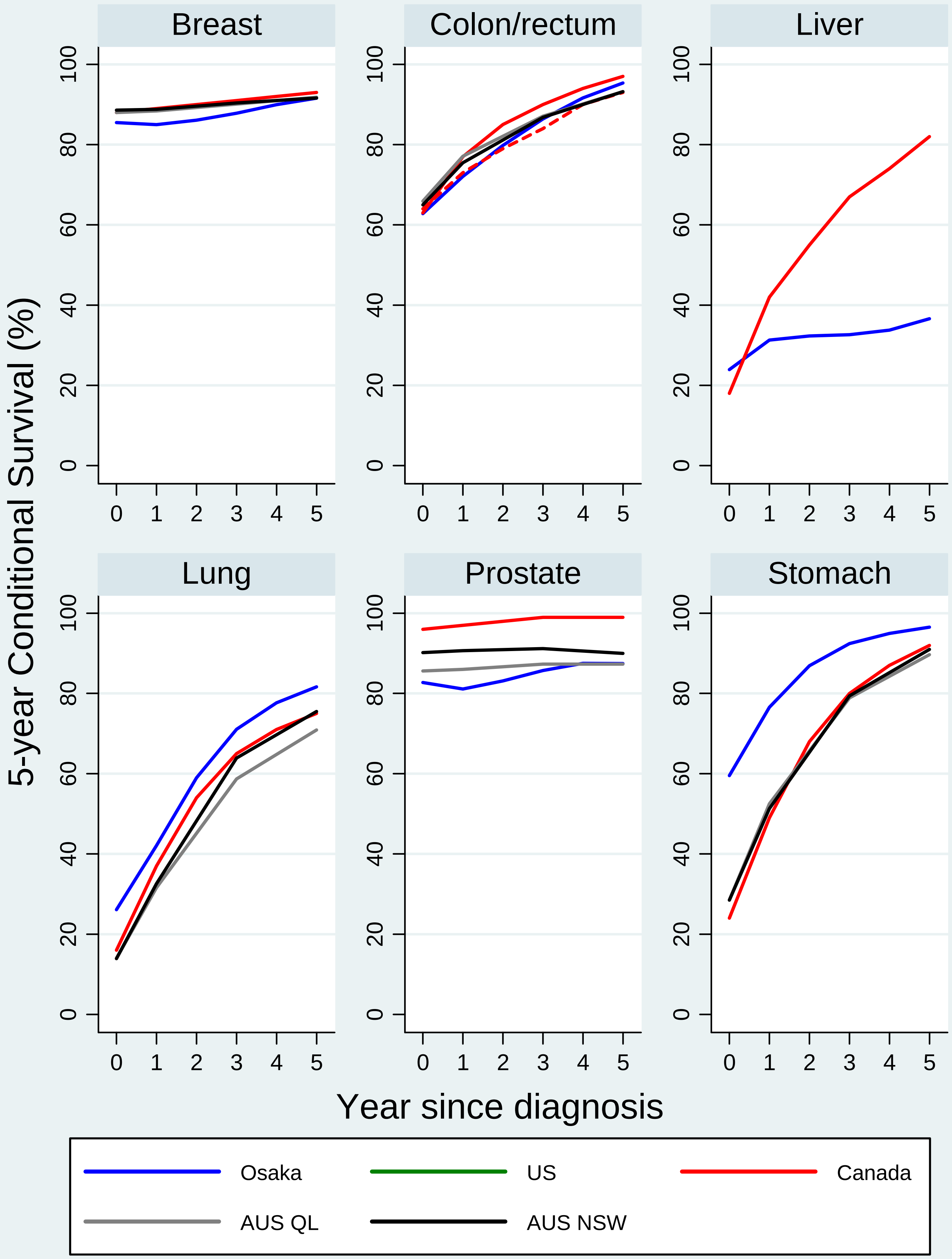

Figure S1. Comparison of five-year conditional survival between countries: All stage

In the Canada colorectal cancer the green solid line represents the colon and the green dotted line represents the rectum.

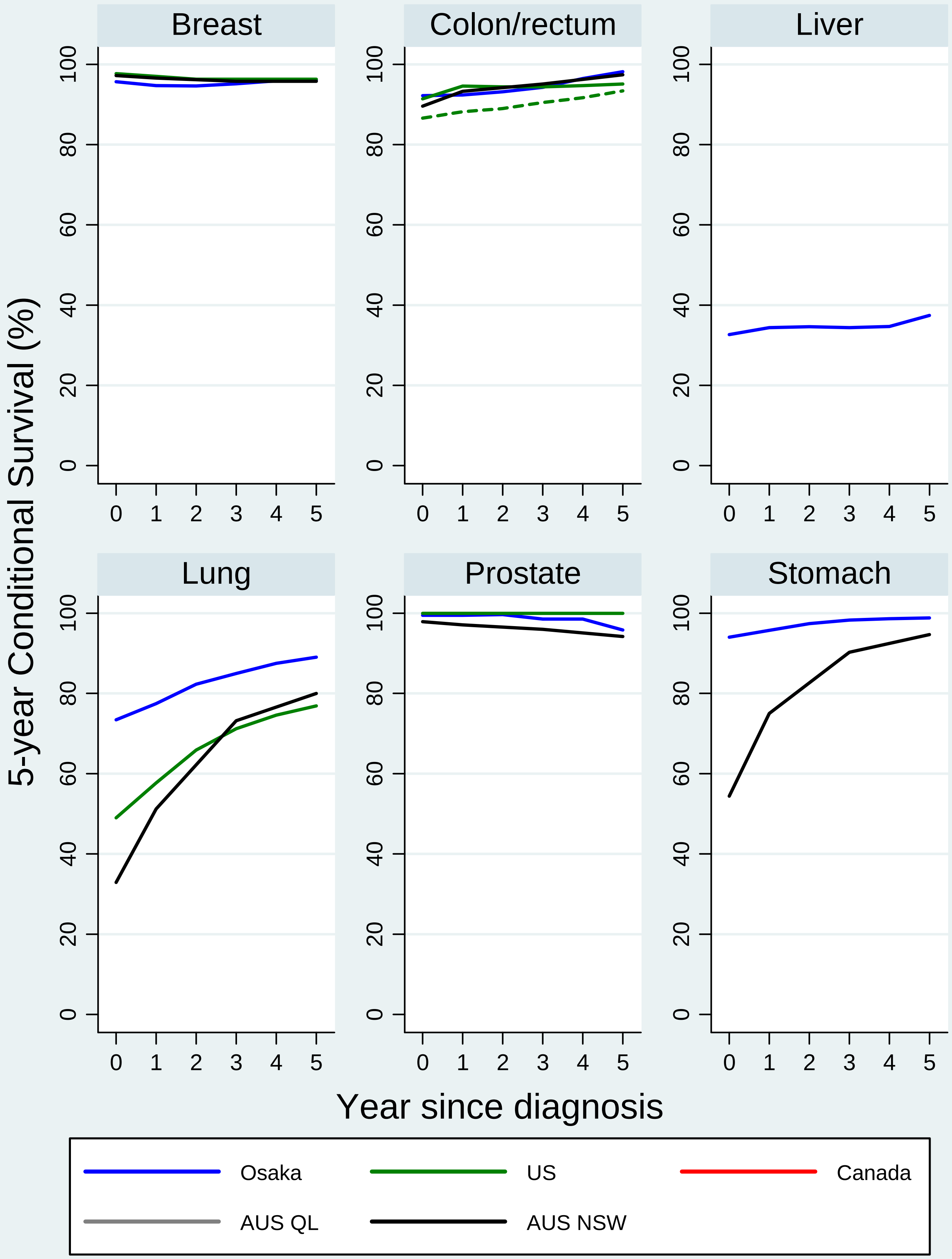

Figure S2. Comparison of five-year conditional survival between countries: Localised

In the US colorectal cancer figures the red solid line represents the colon and the red dotted line represents the rectum.

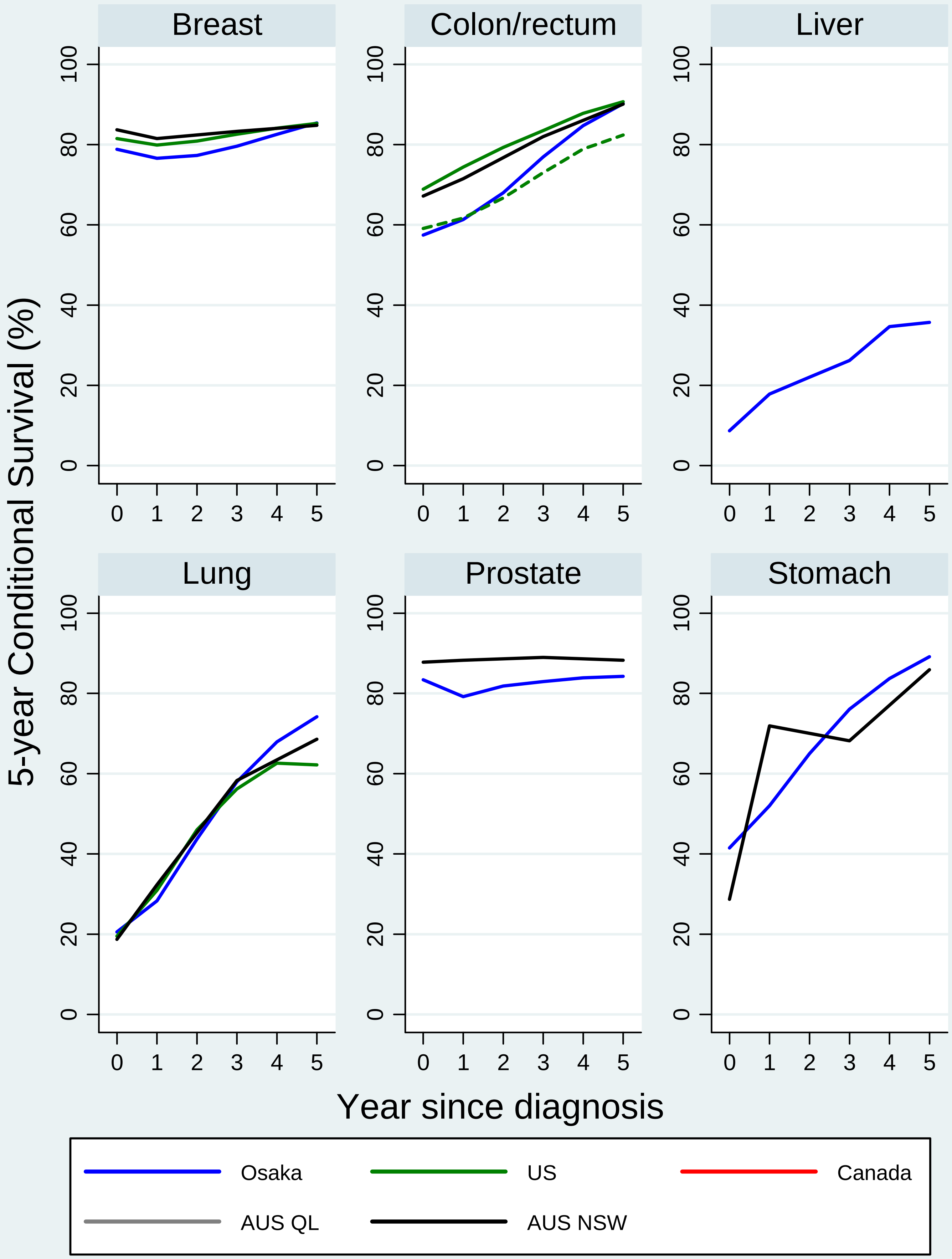

Figure S3. Comparison of five-year conditional survival between countries: Regional

In the US colorectal cancer figures the red solid line represents the colon and the red dotted line represents the rectum.

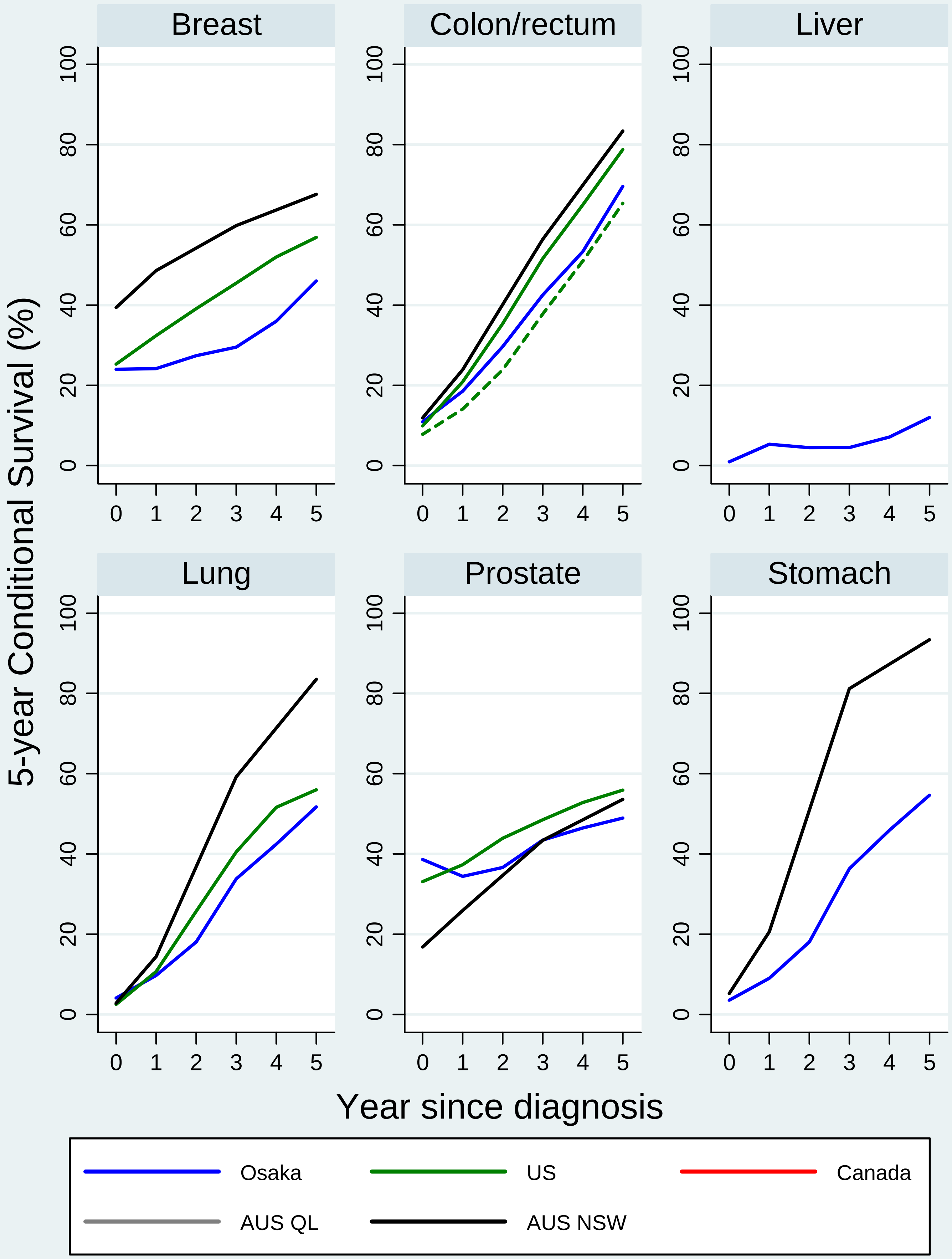

Figure S4. Comparison of five-year conditional survival between countries: Distant

In the US colorectal cancer figures the red solid line represents the colon and the red dotted line represents the rectum.
